# NAT10-mediated ac4C RNA acetylation stabilizes CXCL5/DEK mRNA to drive proliferation and metastasis in lung adenocarcinoma

**DOI:** 10.1038/s41419-026-08568-6

**Published:** 2026-03-20

**Authors:** Xin Hu, Meiqi Feng, Chengjin Qi, Boran Li, Hongjuan He, Kai Li, Lu Chen, Boshu Ji, Haoran Yu, Yue Zhao, Tong Wu, Ruiheng Ma, Yuhao Dong, Yan Zhang, Qiong Wu

**Affiliations:** 1 School of Life Science and Technology, Faculty of Life Science and Medicine, Harbin Institute of Technology, Harbin, 150001 Heilongjiang China; 2 Department for Biomedical Research Bern (DBMR), University of Bern, Bern, Switzerland; 3 School of Medicine and Health, Harbin Institute of Technology, Harbin, 150001 Heilongjiang China; 4 Department of Pathology, the Second Affiliated Hospital of Harbin Medical University, Harbin, 150081 Heilongjiang China; 5 Department of Biomedical Engineering, School of Medical Information and Engineering, Guangdong Pharmaceutical University, Guangzhou, 510006 Guangdong China; 6 Department of Urology, Xiang’an Hospital of Xiamen University, Xiamen, 361000 Fu Jian China; 7 Harbin Medical University, Harbin, 150081 Heilongjiang China

**Keywords:** Cancer epidemiology, Epigenetics

## Abstract

Investigating the epigenetic mechanisms underlying lung adenocarcinoma (LUAD) through the lens of *N*4-acetylcytosine (ac4C) modification could innovate cancer treatment strategies and targets. We used biological information methods to analyze shared data, with a focus on studying *N*-acetyltransferase 10 (NAT10), which is the only known ac4C “writer” protein. Our analysis revealed a significant upregulation of NAT10 expression in LUAD, a finding that was corroborated by investigations in both LUAD cancer tissue samples and cell lines. Subsequently, we employed CRISPR/Cas9 technology to knock out the NAT10 gene and analyzed the resulting knockout cells using acRIP-seq and RNA-seq techniques. Our findings demonstrated different expressions of the genes C-X-C motif chemokine ligand 5 (CXCL5) and DEK proto-oncogene (DEK), and functional enrichment analysis indicated a strong association with the adhesion signaling pathway. Laboratory experiments revealed that NAT10 acts as an ac4C “writer,” promoting the acetylation of *CXCL5* and *DEK* and thus preventing the degradation of their mRNAs. Moreover, NAT10 was found to significantly affect the number of metastases and tumor growth following the injection of cancer cells into the tail vein of mice. Our research data suggests that targeting NAT10 has the potential to serve as a diagnostic biomarker or prognostic target for developing anti-metastatic therapies aimed at disrupting the adhesion process.

## Introduction

As the leading type of cancer globally, lung cancer ranks among the top in terms of newly confirmed cases and deaths. Lung cancer can be divided into small-cell lung cancer and non-small-cell lung cancer. Among them, non-small cell lung cancer can be classified into lung adenocarcinoma (LUAD) and lung squamous cell carcinoma according to histological morphology [1]. LUAD patients account for about 55% of the total number of lung cancer patients and have become the largest subtype of lung cancer [2]. While there has been a significant improvement in outcomes due to molecular targeted therapies and immunotherapies [3]. For this reason, grasping the development of LUAD and recognizing treatment targets is vital for enhancing patient outcomes.

Recent studies underscore that proliferation is a central driver of lung adenocarcinoma (LUAD) aggressiveness and a key determinant of patient outcome [4, 5]. *N*-acetyltransferase 10 (NAT10) has been indicated to be closely associated with malignant proliferation in many types of cancer. In clear-cell renal cell carcinoma, NAT10-driven N4-acetylcytosine (ac4C) on ankyrin repeat and zinc finger peptidyl tRNA hydrolase 1 (ANKZF1) mRNA enhances Yes1- associated transcriptional regulator (YAP1) nuclear retention to accelerate cell-cycle progression and lymphangiogenesis [6]. In hepatocellular carcinoma, ac4C depositions on high mobility group box 2 (HMGB2) and heat shock protein 90 alpha family class A member 1 (HSP90AA1) transcripts boost their translational efficiency via eukaryotic translation elongation factor 2 (eEF2) recruitment, sustaining rapid proliferation and Lenvatinib resistance, while the NAT10 inhibitor-Remodelin restores drug sensitivity [7]. Head-and-neck squamous cell carcinoma exploits ac4C to stabilize glycosylated lysosomal membrane protein (GLMP) mRNA, hyper-activating MAPK/ERK signaling and shortening tumor-doubling time, and knockout of NAT10 reduces Ki67 index by 45% [8]. Likewise, pancreatic ductal adenocarcinoma shows selective ac4C enrichment on AXL receptor tyrosine kinase (AXL) mRNA, which elevates AXL protein levels and drives aggressive proliferation while NAT10 silencing decreases colony formation by 70% [9]. Therefore, it is crucial to investigate the impact of NAT10 on tumor proliferation in LUAD.

Tumor cell adhesion and metastasis are the core aspects of malignant tumor progression, which directly determine tumor invasiveness, metastatic ability and patient prognosis. DEK proto-oncogene (DEK) is an RNA binding protein, which can modulate chromatin accessibility and histone acetylation [10, 11]. In B-cell lymphoma, DEK regulates B-cell proliferative capacity and promotes tumor invasiveness, reducing overall survival [12]. In breast cancer, DEK can promote lymph node metastasis and induce metastasis-related pathways to advance the course of tumors [13, 14]. In cervical cancer, DEK promotes the biological processes of cervical cancer cells through the DEK/β-catenin pathway, accelerating tumor occurrence and metastasis [15]. CXCL5 is a key gene for CXC chemokines that can influence tumor progression by modulating immune responses [16, 17]. CXCL5 promotes tumor cell metastasis by triggering immune cell migration and promotes the immunosuppressive properties of the tumor microenvironment [18]. In gastric cancer, macrophage-released CXCL5 activates the C-X-C motif chemokine receptor 2 (CXCR2) signaling axis under TNF-α-inducing conditions to promote gastric cancer growth and metastasis [19]. In colorectal cancer, CXCL5 can also induce epithelial-mesenchymal transition and promote tumor metastasis through the ERK/AKT pathway [20]. In lung cancer, mouse adult Schwann cells express CXCL5, which activates the PI3K/AKT pathway, increasing the motility phenotype of tumor cells and promoting metastasis [21]. In ovarian cancer, serpin family E member 1(PAI-1) induced the secretion of CXCL5 induce metastasis of cancer cells in feedback loops [22].

Acetylation modification of RNA was first found in tRNA and rRNA, and in 2018, it was confirmed that ac4C exists in eukaryotic mRNA [23]. Since then, RNA acetylation has gradually become an important research object. As a histone acetyltransferase, NAT10 is the only protein that can “write” the acetylation modification of RNA [23]. The acetylation of ac4C is directly involved by acetyl coenzyme A-produced acetyl groups and is energized by ATP/GTP hydrolysis [24]. It has been demonstrated that NAT10 exhibits high levels of expression in many cancers, including hepatocellular carcinoma [24], laryngeal carcinoma [25], and colorectal carcinoma [26], among others. NAT10-mediated modification of mRNA ac4C affects mRNA stability and thus promotes the progression of a wide range of tumors. However, the mechanism of how the ac4C writer NAT10 affects the adhesion and metastasis of lung cancer cells is unknown.

Based on our research, we found that LUAD exhibited elevated levels of NAT10 expression, which were associated with poorer patient outcomes. Through RNA sequencing, acRIP sequencing, and various functional assays, we demonstrated that NAT10 specifically targets *CXCL5* and *DEK*. This targeting facilitates an ac4C modification that helps prevent the degradation of the mRNAs for two genes. Moreover, knocking out NAT10 significantly inhibited LUAD cell proliferation, migration, invasion, and adhesion capabilities in LUAD cells. Notably, the above influence could be reversed by the overexpression of NAT10, CXCL5 and DEK. Additionally, the NAT10-mediated modifications of *CXCL5* and *DEK* also demonstrated the ability to suppress tumor metastasis and proliferation in animal experiments. Our results underscore the major role of NAT10-mediated ac4C RNA acetylation in regulating the adhesion functions of tumor cells. NAT10 can serve as an expected therapeutic target in strategies aimed at combating LUAD metastasis by disrupting the adhesion abilities of tumor cells.

## Results

### NAT10 can serve as a potential marker for LUAD

**Fig. 1:**
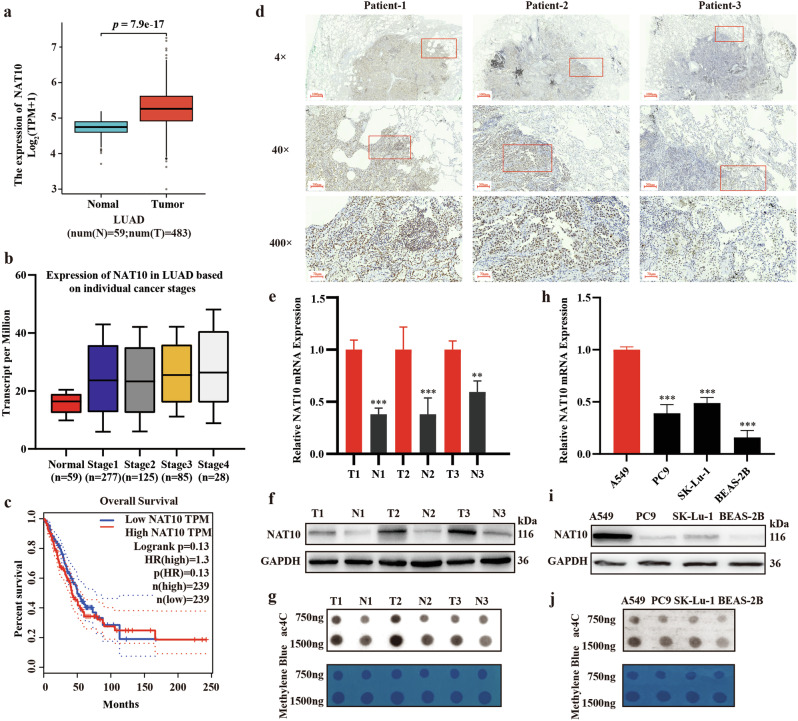
NAT10 can serve as a potential marker for LUAD. **a**, **b** The expression of NAT10 in LUAD cancer tissues and adjacent tissues and its relationship with tumor staging from TCGA. **c** Kaplan–Meier studied LUAD patients with high expression have shorter survival times. **d** NAT10 was examined through immunoblotting in tissue samples from three patients. **e**, **f** Detecting the mRNA and protein expression differences of NAT10 between cancer and adjacent tissues in LUAD patients. **g** Detecting the RNA acetylation level differences of NAT10 between cancer and adjacent tissues in LUAD patients. **h**–**j** Detecting differences in NAT10 expression and RNA acetylation levels between three LUAD cell lines and normal lung epithelial cell lines. The data in (**e**) was normalized to tumor tissue levels. The data in (**h**) was normalized to A549 levels.

RNA acetylation is written by RNA acetyltransferase-NAT10, and the imbalance of modifications may have an impact on the occurrence and development of cancer [23]. To understand how ac4C modification affects LUAD, we analyzed the gene expression of NAT10 using RNA-seq data from The Cancer Genome Atlas Program (TCGA). The findings indicated that NAT10 levels were notably higher in LUAD tumors than in the paracancerous tissues, and the expression increases with stage progression (Fig. 1a, b). Overall survival analysis shows that lung adenocarcinoma patients with high expression have shorter survival times (Fig. 1c). Next, we used immunohistochemical technology to analyze tumor sections of lung adenocarcinoma patients, and the results indicated that NAT10 is mainly expressed in the nucleus of tumor cells rather than in normal alveoli (Fig. 1d). In addition, we detected the expression of NAT10 mRNA and protein in three pairs of LUAD samples and three LUAD cell lines. The data show that the level of NAT10 in cancer tissues of LUAD patients is significantly higher than that in adjacent tissues (Fig. 1e, f). In addition, the results of ac4C dot blot analysis showed a significant increase in overall ac4C levels, which corresponds to the elevated expression of NAT10 in LUAD tissues (Fig. 1g). When compared to the normal lung epithelial cell line BEAS-2B, NAT10 was also found to be significantly overexpressed in the A549, PC9, and SK-Lu-1 LUAD cell lines at the mRNA, protein, and ac4C levels, aligning with the results observed in LUAD tissues (Fig. 1h–j). Due to the highest expression of A549 cells in the above tests, they were selected as the Reverse Genetics experimental subjects for subsequent research.

### NAT10-knockout affects the expression of genes related to cell adhesion

**Fig. 2:**
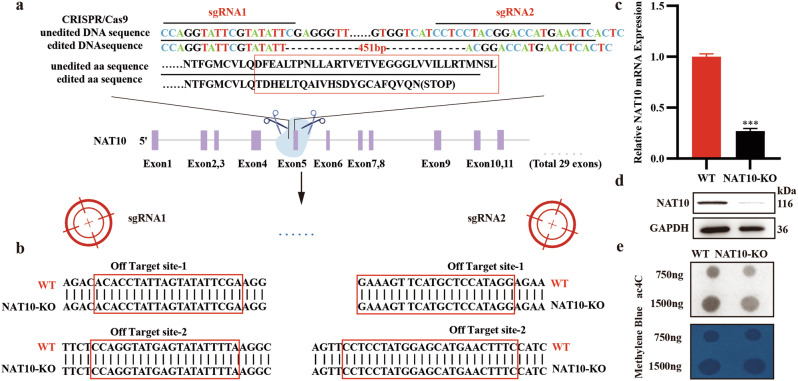
Establishment and detection of NAT10 knockout A549 cell line. **a** Diagram showing how CRISPR/Cas9 is used to delete the NAT10 gene, along with the process of identifying deletion clones of the NAT10 gene through sequencing. **b** The top two off-target sites of each sgRNA were selected for off-Target detection. **c**, **d** In cells where NAT10 has been knocked out, levels of NAT10 expression were significantly lower. **e** ac4C dot blot between the wild-type A549 cell line and the NAT10 knockout A549 cell line. The data in (**c**) was normalized to WT levels. The model of CRISPR/Cas9 in A was created in https://BioRender.com.

Revealing the target genes mediated by RNA acetylation modification of NAT10 is an important step in exploring the impact of NAT10 on tumor progression. Firstly, we employed the CRISPR/Cas9 technology to create a stable NAT10-knockout cell line in A549. We chose the best single guide RNA by the Benchling website (https://www.benchling.com). In this study, these two single guide RNA sequences targeting the shared fifth exon of all transcripts are shown in Fig. 2a. Using Primer5.0, external primers were designed at both ends of the knockout site to identify the knockout, and internal primers were designed at the inner side of the knockout site to identify whether it was a homozygote. PCR results showed that the knockout band was 367 bp and there was no internal primer to amplify the bands, so we obtained a homozygote clone with NAT10 knockout (Fig. S1). The top two off-Target sites of each sgRNA were selected for off-target detection in the above cell lines, and sequencing results indicated that neither sgRNA produced off-Target (Fig. 2b). The knockout site of NAT10 was examined through sequencing, and the efficiency was checked with quantitative real-time polymerase chain reaction (qRT-PCR) and Western Blot. Compared to wild-type cells, the mRNA and protein expression levels of NAT10, as well as the RNA acetylation modification mediated by it, are also significantly reduced (Fig. 2c–e).

**Fig. 3:**
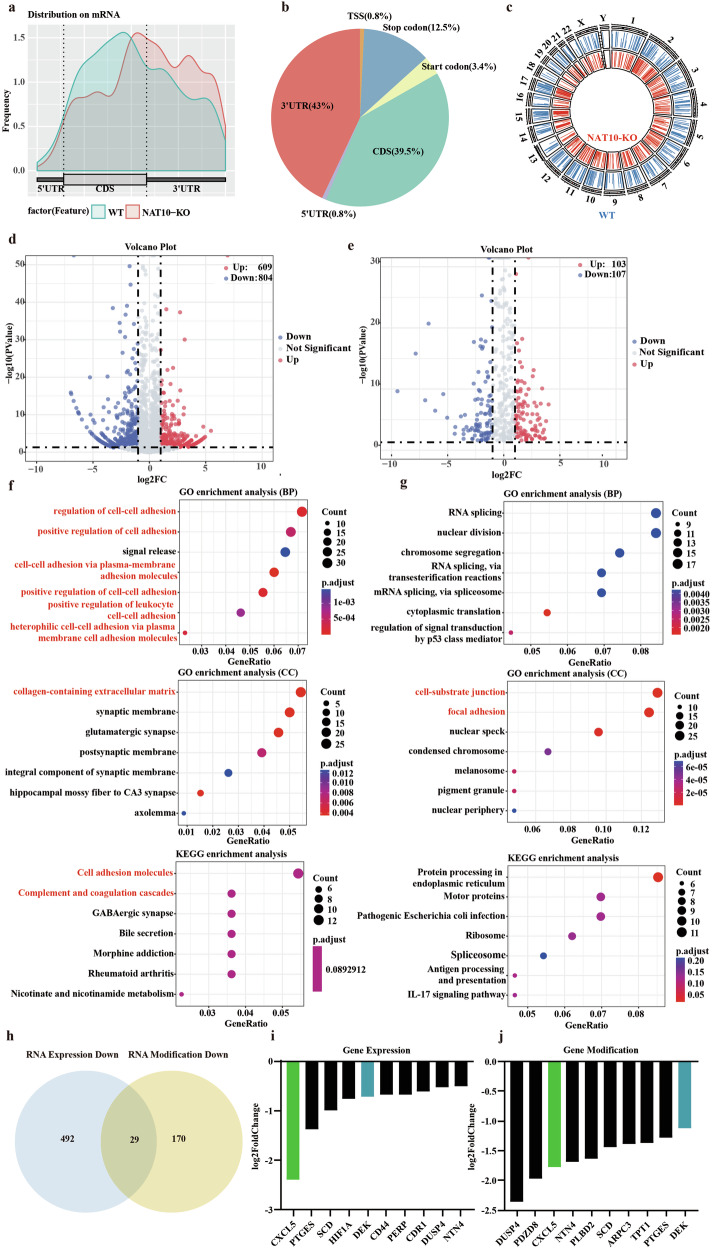
NAT10-knockout affects the expression of genes related to cell adhesion. **a** Due to the knockout of NAT10, the enrichment degree of ac4C modification on mRNA varies across different regions. **b** Proportion of RNA acetylation enriched regions in wild-type A549 cells. **c** The effect of NAT10 knockout on ac4C modification on various chromosomes. **d** RNA-Seq revealed that NAT10 stable knockout cells have genes expressed at different levels. **e** acRIP-Seq revealed that NAT10 stable knockout cells have genes modified at different levels. **f**, **g** GO functional analysis of downregulated genes through expression and modification, as well as KEGG pathway enrichment results. **h** Intersection of genes with downregulated mRNA expression and downregulated RNA acetylation modification. **i**, **j** Top ten genes with downregulated mRNA expression and downregulated RNA acetylation modification.

Then, to explore the target genes regulated by NAT10 through the ac4C pathway, we performed acRIP-seq and RNA-seq using wild-type and stable NAT10-knockout A549 cells. After knocking out NAT10, the ac4C modification in the 5’UTR and CDS regions of the entire transcriptome mRNA was downregulated, which is consistent with the trend of ac4C modification gradually weakening from 5’UTR to CDS region and then to 3’UTR [23] (Fig. 3a). Figure 3b, c shows that the ac4C modification of wild-type A549 cells mainly exists in the 3’UTR and CDS regions and the effect of NAT10 knockout on ac4C modification on various chromosomes. After knocking out NAT10, the enrichment level of ac4C peaks in the coding sequence (CDS) of A549 cells decreased from 39.5 to 22.2% in acRIP-seq data. The most significant downregulation of ac4C modification on chromosome 11 after NAT10 which is located on chromosome 11 knockout demonstrated the possibility that NAT10 tends to modify nearby sites (Fig. S2).

After knocking out NAT10, the analysis of RNA sequencing revealed that there are 804 genes which have significantly decreased expression, while 609 genes have shown increased expression (Fig. 3d). Analysis of acRIP-seq revealed that modifications in 107 genes were significantly reduced, while modifications in 103 genes were increased (Fig. 3e).

At present, most literature reports that NAT10 knockdown will lead to the downregulation of ac4C modification of target genes, and will reduce the expression of target genes by reducing the stability of target genes [27–30]. We focused on genes with downregulated gene expression and RNA acetylation modification. Gene Ontology (GO) analysis (biological processes and cellular components) (*p* < 0.05) and Kyoto Encyclopedia of Genes and Genomes (KEGG) pathway clearly indicate that NAT10 has the potential to affect biological processes related to tumor metastasis (*p* < 0.2), such as cell adhesion and extracellular matrix containing collagen (Fig. 3f, g). According to acRIP-seq and RNA-seq, 29 genes whose expression and modification were downregulated after NAT10 knockout were taken as the intersection (Fig. 3h). Figure 3i, j show the top ten genes with downregulation of expression and modification, of which *CXCL5* and *DEK* are among them. Moreover, several literatures have reported that CXCL5 and DEK are closely related to tumor metastasis and adhesion in a variety of cancers [10, 12, 16, 20], which also verified the previous results of GO function and KEGG enrichment. When considering all the results mentioned, it appears that NAT10 might control the CXCL5/ DEK levels.

### Identified CXCL5 and DEK as targeted regulation genes by NAT10-mediated ac4C modification, enhancing their mRNA stability

**Fig. 4:**
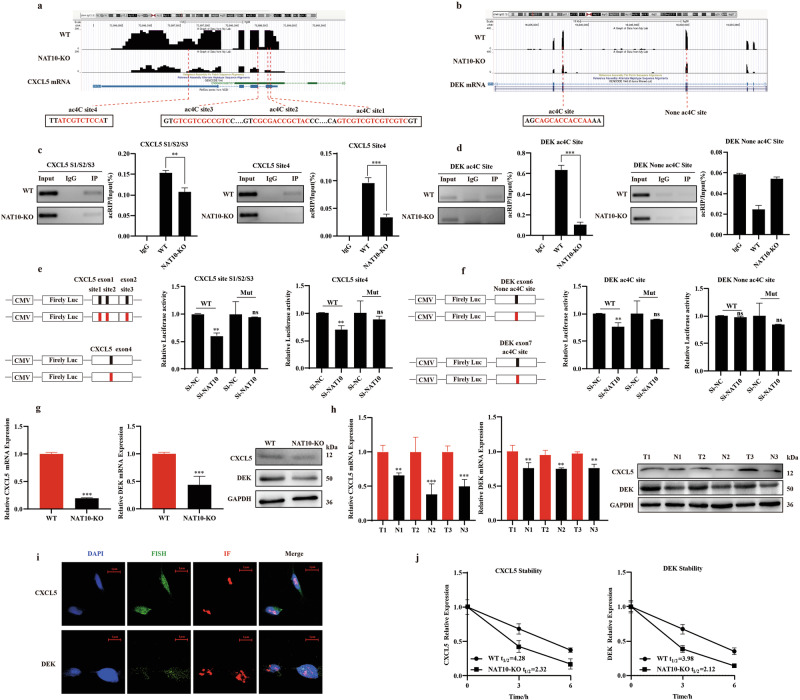
Identified *CXCL5* and *DEK* as targeted regulation genes by NAT10-mediated ac4C modification, enhancing their mRNA stability. **a**, **b** Distribution of ac4C modification sites on mRNA of CXCL5 and DEK. **c**, **d** The ac4C modification of *CXCL5* and *DEK* was verified through acRIP-PCR. **e**, **f** The dual fluorescence experiment confirmed the importance of NAT10 recognizing the ac4C modification site of the target gene. **g**, **h** Detecting the differential mRNA and protein levels of *CXCL5* and *DEK* before and after NAT10 knockout, as well as cancer tissues and adjacent tissues in LUAD. **i** Co-localized the intracellular expression of NAT10 with *CXCL5* or *DEK* by Fish and IF. **j** The half-life changes of CXCL5 and DEK mRNA after inhibiting NAT10. The data in (**e**, **f**) were normalized to Si-NC levels. The data in (**g**) was normalized to WT levels. The data in (**h**) was normalized to tumor tissue levels. The clinical samples used in Fig. 4h is consistent with those in Fig. 1f.

As the current literature reports that ac4C modification usually exists on the repeat sequence with “Cxx..Cxx..Cxx..Cxx” on mRNA [30–32], combined with the ac4C peak data of acRIP-seq, there are three ac4C modification sites on the first and second exons of *CXCL5* and one ac4C modification site in the fourth exon, a peak without “Cxx..Cxx..Cxx..Cxx” repeat sequence in the sixth exon of *DEK* and one ac4C modification site in the seventh exon (Fig. 4a, b). To explore if NAT10 affects the mRNA of *CXCL5* and *DEK* through RNA acetylation modification, we carried out acRIP-PCR to check the presence of ac4C in *CXCL5* and *DEK*. The acRIP experiment demonstrated that both ac4C modification sites on *CXCL5* mRNA were downregulated with the downregulation of NAT10. The sites with repetitive sequences on *DEK* mRNA also conform to this regulatory mechanism, while the sites without repetitive sequences do not (Fig. 4c, d). At the same time, the negative control used a gene without ac4C modification sites, which further proves the reliability of the experiment (Fig. S3).

To better understand how ac4C modification of the target mRNA influences gene regulation, we conducted specific experiments. We created constructs with either normal or mutated *CXCL5* and *DEK* ac4C sites, then evaluated how the ac4C modification affected the expression of *CXCL5* and *DEK* using a luciferase reporter assay. In the mutant *CXCL5* and *DEK* constructs, the cytosine bases (C) in the ac4C consensus sequence were replaced by guanine (G), successfully removing the ac4C modification. As expected, luciferase reporter assays showed a significant reduction in ac4C modification in wild-type *CXCL5* and *DEK* compared to the mutant in NAT10 knockdown (siNAT10) (Fig. 4e, f). Our results also showed that the mRNA and protein expressions of these two genes were significantly downregulated after knockout of NAT10, which was also verified in lung adenocarcinoma tissues (Fig. 4g, h).

In addition, we also co-localized the intracellular expression of NAT10 with *CXCL5* or *DEK* by RNA-Fish and Immunofluorescence (IF) (Fig. 4i). Furthermore, we calculated the half-life of RNA in the NAT10 knockout A549 cell line and wild-type A549 cell line. Upon NAT10 knockout in A549 cells, we observed a decreasing trend in the levels of *CXCL5* and *DEK* mRNAs, and the half-life of both mRNA molecules continued to be significantly shortened (Fig. 4j). Based on these results, we hypothesized that *CXCL5* and *DEK* are the targets of NAT10 and their expression regulation is dependent on NAT10-mediated ac4C modification.

### ac4C modification of CXCL5/DEK promotes tumor adhesion in LUAD cells in vitro and in vivo

**Fig. 5:**
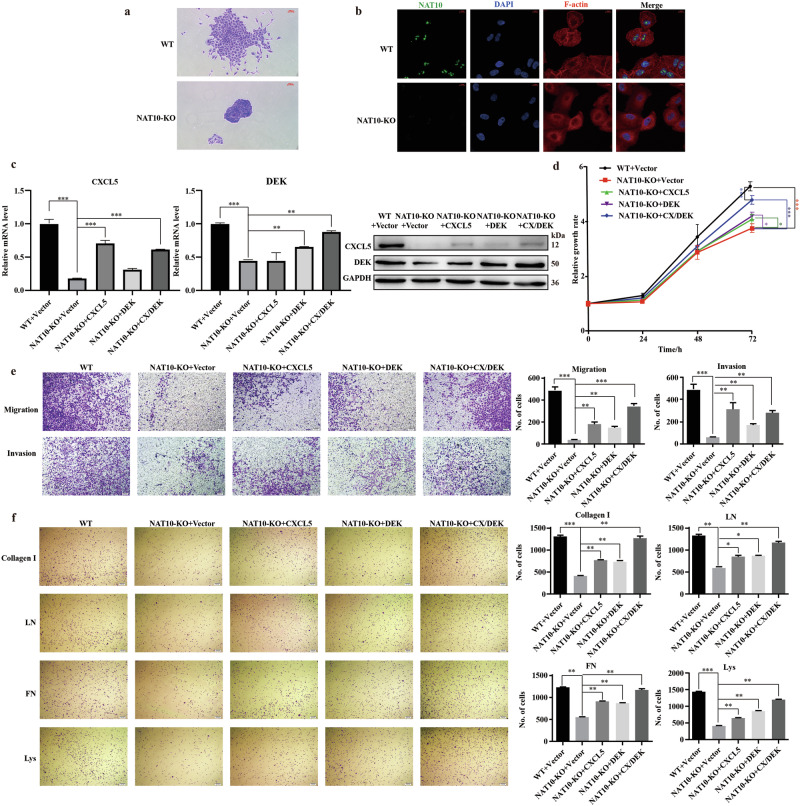
ac4C modification of *CXCL5*/*DEK* promotes adhesion in LUAD cells in A549 cell. **a** Phenotypic comparison of A549 wild-type and NAT10-knockout cells. **b** Co-localized the intracellular expression of NAT10 with F-actin by IF. **c** Prove the effect of overexpression of CXCL5 and DEK separately or simultaneously at the mRNA and protein levels. **d** Cell proliferation was measured using the CCK8 assay after NAT10 knockout and CXCL5/DEK overexpression. **e** Inhibition of invasion and metastasis of LUAD cells by NAT10 and its rescue effect on CXCL5/DEK. **f** Inhibition of cell adhesion of LUAD cells by NAT10 and its rescue effect on CXCL5/DEK. The data in (**c**) was normalized to WT + Vector levels.

We observed the phenotypes of wild-type A549 cells and NAT10 knockout A549 cells by cell colony formation and found that the wild-type cell clusters were looser while the knockout group was tighter. It was hypothesized that the wild-type cell motility was significantly stronger than the NAT10 knockout cell line (Fig. 5a). Then, we used immunofluorescence to verify whether the altered cell motility was associated with intracellular cytoskeletal changes (Fig. 5b). When NAT10 was absent, the location of cytoskeletal proteins labeled by F-actin changed, and a large number of proteins retracted around the nucleus, from which it can be inferred that NAT10 influences the cytoskeleton thereby directly regulating cell motility [33, 34].

To investigate whether two target genes have the same promoting effect on lung adenocarcinoma cell adhesion as NAT10. Based on this, we upregulated the expression levels of CXCL5 and DEK using the PCDNA3.1 overexpression vector, aiming to evaluate the promotional role of NAT10 and its target genes, *CXCL5* and *DEK*, in tumorigenesis and development. We have demonstrated good overexpression effects of two target genes by detecting mRNA and protein expression levels. (Fig. 5c). Cell proliferation experiments have shown that the proliferation ability of A549 cells is weakened after NAT10 knockout, while overexpression of CXCL5 or DEK partially restored the cell proliferation. Further studies revealed that cells co-expressing CXCL5 and DEK in the context of NAT10 knockout showed significantly faster proliferation compared to cells overexpressing CXCL5 or DEK alone (Fig. 5d).

Next, we evaluated the effects of NAT10 on LUAD cells' migration and invasion. The experimental results revealed the migration and invasion ability of LUAD cells after NAT10 knockout. In addition, overexpression of CXCL5/DEK alone or in combination has a restoring effect on the impact of NAT10 knockout (Fig. 5e).

Cell adhesion is a necessary process in tumor metastasis, and its enhanced adhesion ability to extracellular mechanisms makes it easier for tumor cells to form metastatic foci, which is a risk factor for tumor metastasis. Therefore, to explore the effect of NAT10-mediated CXCL5/DEK in LUAD adhesion, we used four kinds of adhesive Matrigel: Collagen I, laminin (LN), fibronectin (FN) and polylysine (Lys). As shown in Fig. 5f, NAT10 deletion significantly inhibited cell adhesion, whereas CXCL5/DEK overexpression can significantly restore LUAD cell migration and invasion.

Meanwhile, we further evaluated the impact of NAT10 rescue on the phenotype of cells after NAT10 knockout. We utilized the PCDNA3.1 overexpression vector to upregulate the expression level of NAT10. The results indicate that the overexpression of NAT10 can rescue the effects brought about by NAT10 knockout (Fig. S4a–c). To further confirm the importance of the acetylation catalytic site of NAT10 in the regulation process of target genes, since some research has demonstrated that the G641E site on NAT10 is crucial for catalyzing the acetylation site [35–38], we mutated the G641E site on the NAT10 overexpression vector. However, when NAT10-G641E was overexpressed in both wild-type and NAT10 knockout cells, there were no significant changes in the mRNA levels of *CXCL5*/*DEK*, which validates the previous researchers’ conclusion that NAT10 catalytic activity is crucial for the regulation of target genes (Fig. S4d).

**Fig. 6:**
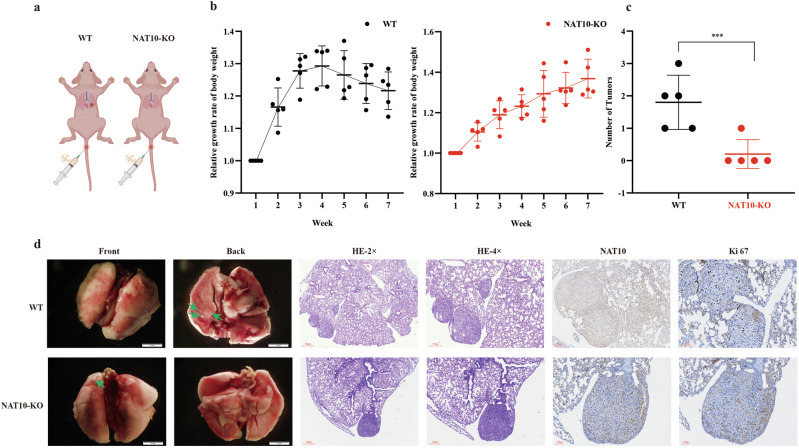
NAT10 promote metastasis in LUAD cells in vivo. **a** Schematic diagram of mouse tail vein injection. **b** Injecting NAT10 deficient A549 cells and a control group into the tail vein of nude mice to observe changes in their body weight. **c** Number of lung metastases in nude mice (*n* = 5). **d** Representative photos of lung metastases in nude mice. Representative images of IHC and HE staining for NAT10 and Ki67 protein on a tumor tissue. **a** was created in https://BioRender.com.

Cell experiments have demonstrated the inhibitory effect of NAT10 and its target genes on lung adenocarcinoma cells. We plan to demonstrate the specific effect of NAT10 knockout on lung adenocarcinoma tumor metastasis in nude mice. We injected wild-type A549 cells and NAT10 stable knockout A549 cells into the tail vein of nude mice to observe the in vivo effects of NAT10 (Fig. 6a) and weighed the body weight of the mice every 3 days. As shown in Fig. 6b, c, the weight gain rate of mice in the NAT10 knockout group was significantly higher than that in the control group, and the number of metastatic lesions formed after 7 weeks was significantly lower than that in the control group. Hematoxylin-eosin staining (HE) and immunohistochemistry (IHC) also demonstrated that NAT10 knockout could inhibit the metastasis of LUAD and the proliferation of LUAD in vivo through the expression of Ki67 (Fig. 6d).

Our data support that NAT10 promotes the proliferation and metastasis of lung adenocarcinoma by mediating acetylation modification of target genes and enhancing mRNA stability of target genes.

## Discussion

**Fig. 7:**
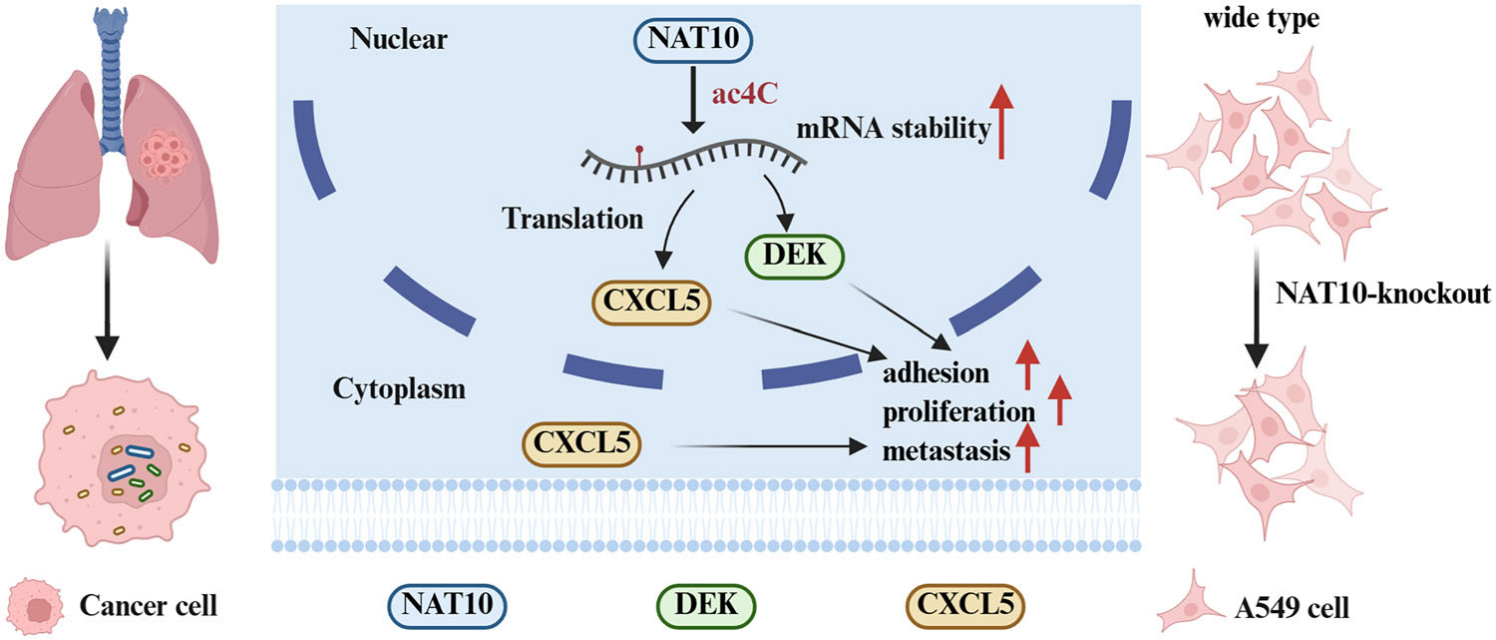
The potential mechanism model was constructed in this study. Initially, NAT10 acetylates CXCL5 and DEK to maintain their mRNA stability and expression. Then, the increased expression of CXCL5 and DEK promoted tumor adhesion to facilitate LUAD proliferation and metastasis. Created in https://BioRender.com.

*N*4-acetylcytidine acetylation is an acetylation modification present on RNA molecules that is catalyzed by the only acetyltransferase-NAT10 [23]. Studies have revealed that NAT10 plays diverse pro-oncogenic roles in different cancer types. In colorectal cancer, NAT10 influences the advancement of colorectal cancer by controlling how stable S100 calcium-binding protein A4 (FSP1) mRNA is and impacting the processes related to ferritinase [27]. Research shows that in breast cancer, NAT10 modifies MORC family CW-type zinc finger 2 (MORC2) by acetylation. This process plays a key role in controlling cell-cycle checkpoints, which affects how cells respond to chemotherapy and radiotherapy that can damage DNA [39]. In multiple myeloma, the protein NAT10 plays a key role in increasing how well CEP170 mRNA is translated by adding acetyl groups to it. This process leads to the growth of multiple myeloma cells [40]. In this study, we indicate that in LUAD, upregulation of NAT10 expression levels triggers aberrant ac4C modification phenomena, and that acetylation of *CXCL5* and *DEK* by NAT10 induces tumor cell adhesion and proliferation to promote LUAD progression (Fig. 7).

Disrupting the adhesion of tumors is crucial for inhibiting the occurrence and progression of cancer. In breast cancer, branched-chain keto acid dehydrogenase kinase (BCKDK) regulates cell adhesion and tumor metastasis by blocking the ubiquitination modification pathway of talin1 by tripartite motif containing 21 (TRIM21) [41]. Circulating tumor cells induce platelet activation and aggregation and promote cancer metastasis [42]. Breast cancer cells cause MSCs to release C-C motif chemokine ligand 5 (CCL5), which influences the cancer cells and increases their movement, ability to invade, and spread to other areas [43]. NAT10 is the sole molecule that possesses acetyltransferase activity, enabling it to modify RNA with ac4C. To further investigate this, we utilized CRISPR-Cas9 to reduce NAT10 gene levels in A549 cells. Then, we compared gene expression levels between the knockdown group and the wild-type A549 cells using RNA-seq and acRIP-seq methods. Through acRIP-PCR and luciferase reporter assays, we found that 804 genes showed significant differences in expression and were mainly related to cell adhesion and growth functions. Meanwhile, among the ten DEGs with downregulated expression, we successfully identified the best ac4C motif sites for CXCL5 and DEK. It is shown that *CXCL5* and *DEK* are important functional targets of NAT10.

The process of adding an acetyl group to the coding sequence of mRNA improves its stability and how well it gets translated, which in turn boosts gene expression. [23]. In addition, ac4C acetylation modification of RNA can provide richer transcript types by promoting variable splicing and secondary structure formation of RNA [44]. Our data showed that the RNA decay rate was the same in the NAT10 knockout A549 cell line and the wild-type A549 cell line. After NAT10 knockout in A549 cells, *CXCL5* and *DEK* mRNA levels began to decrease, and the half-life of these mRNA molecules continued to shorten significantly.

Cancer is defined as a collection of abilities that cells gain as they progress to a tumor stage. These abilities include the ability to keep signals for growth, become able to replicate indefinitely, and start the process of spreading and invading other tissues [45]. The family of integrins, which are receptors for cell adhesion, can influence various activities of many cancer cells, including their movement, ability to invade, growth, and survival. These activities are crucial in cancer development, growth, and spread. Research has indicated that certain integrins are closely linked to the worsening of the disease and decreased survival rates for patients across various types of tumors [46]. This research uncovered that acRIP-seq, RNA-seq, and the roles of specific genes all indicate that adhesion is significant. We found that CXCL5/ DEK was acetylated by NAT10, and CXCL5/DEK promotes the adhesion and growth of tumor cells. This was validated through both laboratory and live organism experiments.

In summary, through laboratory and animal studies, we have firmly established the important role of ac4C modification mechanism in the development of LUAD. Further research suggests that NAT10 can recognize CXCL5 and DEK through RNA acetylation modification, which works together to enhance tumor cell adhesion and growth, thereby revealing the molecular processes of tumor formation and progression. This study aims to provide potential diagnostic markers or targets for prognosis, to assist in the development of anti-tumor drugs that focus on cell adhesion properties.

## Materials and methods

### Clinical samples information

Samples of three pairs of LUAD tissues along with their nearby normal tissue specimens were randomly obtained from the Second Affiliated Hospital of Harbin Medical University.

### Cultivation of LUAD cell lines

The human lung adenocarcinoma cell lines, A549, PC9, and SK-Lu-1, as well as the human lung epithelial cell line BEAS-2B, were obtained from ATCC. These cell lines were grown in DMEM (Gibco, New York, USA) that contained 10% fetal bovine serum (CellMax, Beijing, China) and 1% penicillin-streptomycin (Beyotime, Shanghai, China) at a temperature of 37 °C with 5% CO₂. To maintain their quality, mycoplasma contamination tests were conducted regularly. The above cell lines were identified by STR.

### Extraction of RNA and qRT-PCR

For the extraction of RNA from tissues and cells, we utilized RNAiso Plus. To determine the total RNA concentration, measurements were taken with the NanoUV-Vis spectrophotometer (Thermo, Massachusetts, USA). Following this, cDNA was generated using the PrimeScript^TM^ RT reagent Kit (TaKaRa, Beijing, China), following the instructions provided by the manufacturer. We performed qRT-PCR to evaluate the levels of mRNA expression, using the TB Green Premix Ex Taq (TaKaRa) on the High-productivity Real-Time PCR machine (ABI 7500, USA), with GAPDH acting as the reference gene. The specific primers used for qRT-PCR can be found in Table S1.

### Western blot analysis

Proteins were extracted from tissue or cell samples using RIPA lysis buffer, which included PMSF and a Protease Inhibitor Cocktail (Beyotime, China). The concentration of the proteins was then determined with a BCA kit (Beyotime, China). Next, the proteins were carefully transferred to gels and PVDF membranes for SDS-PAGE analysis. After blocking with 5% skimmed milk, the membranes were incubated overnight at 4 °C with specific primary antibodies, followed by an hour at room temperature with HRP-conjugated secondary antibodies. For detection, an automatic chemiluminescence analysis system (Tanon 5200, China) and an ECL kit (Beyotime, China) were employed. The antibodies used in this process consisted of NAT10 (1:1000, Abcam, ab194297), CXCL5 (1:300, Abcam, ab126763), DEK (1:500, CST, 29812 T), GAPDH (1:1000, Abcam, ab181602), HRP-conjugated secondary goat anti-rabbit IgG (1:5000, Proteintech, SA00001-2), and Goat anti-Mouse IgG (1:5000, ABclonal, AS003).

### ac4C dot blot assays

Total RNA concentration was measured using the NanoDrop instrument. RNA samples after heating at 95 °C for 5 min and cooling to RT, were spotted onto a Hybond-N+ membrane (Invitrogen, California, USA). The membranes were crosslinked, blocked, and incubated with an anti-ac4C antibody (1:1000, Abcam, ab252215) at 4 °C overnight. The membrane was then washed and incubated with an HRP-conjugated secondary antibody for 1 h at room temperature. Signal development method as described above. Methylene blue staining was used to confirm equal loading.

### Immunohistochemical analysis

Tissue sections that had been embedded in paraffin were first treated with xylene to remove any wax. Following this, they went through a rehydration process using a gradient of ethanol solutions. The sections received treatment with sodium citrate buffer and a 30% hydrogen peroxide solution. Afterward, they were incubated with an anti-NAT10 primary antibody, followed by a secondary antibody that was conjugated with HRP. To create a color reaction, diaminobenzidine was applied, and hematoxylin was used for staining the nuclei. Stained sections were then scanned using a digital slide scanner (Easyscan 6, MOTIC), and the images were processed through MOTIC’s dedicated software and network.

### Establishment of stable cell lines with NAT10 knockout, NAT10, CXCL5, and DEK overexpression in A549 cells

To create NAT10-knockout A549 cell lines, two single guide RNAs (sgRNAs) were designed by Benchling (https://benchling.com) with the following sequences: CCAGGTATTCGTATATTCGA and CCTACGGACCATGAACTCAC. These sgRNAs were then annealed and placed into the BbsI and BsaI (NEB, USA) sites of the PX458 vector. Following this, Lipofectamine^TM^ 3000 (Thermo Fisher Scientific, USA) was used to transfect the vector into A549 cells. After undergoing puromycin selection and limited dilution, single-cell clones were isolated. The knockdown efficiency was evaluated, and the stable NAT10 knockout cell line was selected for additional experiments. Details about the primers for CRISPR-Cas9 can be found in Table S2. Additionally, to enhance the expression of NAT10, CXCL5 and DEK, their respective cDNAs were amplified and inserted into the pcDNA3.1-EGFP vector. The construction of the NAT10-G641E mutation was used by the KOD-Plus-Mutagenesis Kit (TOYOBO, SMK-101).

### RNA-seq and acRIP-seq analyses

The total RNA that was extracted was sent to Guangzhou Epibiotek Co., Ltd. in Guangzhou, China, for RNA sequencing. For acRIP-seq analyses, mix total RNA with protein A/G magnetic beads and anti-modification antibodies, break them at high temperature and capture fragments with ac4C modification. After removing rRNA, synthesize the first strand of cDNA, amplify it by PCR, and perform RNA-seq sequencing. The raw image files obtained from high-throughput sequencing must undergo base recognition and error filtering to obtain sequence data. Determine the source of each sequence through genome alignment for visualization in IGV or Genome Browser (UCSC). Peak annotation analysis was performed by clarifying the enrichment of these short sequences in the genome to determine the location and degree of changes in ac4C modification after knocking out NAT10.

This research identified differentially expressed genes (DEGs) using a log₂fold change of under −1 and a *p* value lower than 0.05 (Table S3). The DEGs were used for GO (*p* < 0.05) and KEGG (*p* < 0.2) enrichment analysis. DAVID software was applied to perform Gene Ontology function enrichment analysis for these DEGs, which included cellular components and biological processes.

Furthermore, when identifying genes with both ac4C modification and downregulation of RNA expression, except for ensuring a *p* value lower than 0.05 (Table S4), our threshold for genes with downregulation of RNA expression is log_2_foldchange less than −1 and WT INPUT Count greater than 20 to avoid genes that are difficult to detect in cells. For genes with downregulation of modification, our threshold is log_2_foldchange less than 0 to expand the number of subsequently detectable genes.

The raw data of acRIP-seq (GSE315123) and RNA-seq (GSE315121) have been uploaded to the GEO database.

### acRIP assay and RT-PCR

Cell acRIP assays were conducted in accordance with the instructions provided in the Magna MeRIP™ m6A Kit (Millipore, 17-10,499) and referenced studies [37], with a few modifications. Initially, total RNA at 10 μg was subjected to heating at 94 °C along with 10× Fragmentation buffer. To stop the fragmentation, 2 μL of 0.5 M EDTA was included. This mixture was then diluted to a final volume of 200 μL and the upper phase was extracted using centrifugation with phenol/chloroform. A fresh quantity of chloroform was added, followed by another centrifugation, and the aqueous part was carefully transferred into a new tube. The RNA was precipitated utilizing a solution of linear acrylamide (Sangon Biotech, A610548) and ethanol. After two washes with 75% ethanol, the RNA was dissolved in water that was free of nucleic acids.

Following this, Magnetic Beads coated with Magna ChIP Protein A/G were resuspended in 1×IP buffer. After that, either 0.5 μg of anti-ac4C or anti-IgG was introduced. The beads were washed three times with 1× IP buffer post 30 min at room temperature. For the RIP reaction, the fragmented RNA was combined with IP buffer, nuclease-free water, and an RNase inhibitor and incubated with the magnetic beads coated with either anti-ac4C or IgG while rotating at 4 °C. The magnetic beads were then washed after this step. Next, the beads with the RNA/antibody complex were resuspended in a buffer that included Tris-HCl, NaCl, EDTA, SDS, and proteinase K, and incubated for 30 min at 37 °C. The supernatant was collected, and again phenol/chloroform was added to purify the RNA as previously described.

The purified RNA underwent immediate reverse transcription followed by PCR amplification. The ac4C levels in each RIP group were normalized based on the input group findings. The primers used for acRIP-PCR can be found in Table S5.

### Luciferase reporter assay

Assays using luciferase reporters were conducted in A549 cells. To reduce NAT10 expression, siRNA specifically directed at NAT10 was introduced, while a negative control was kept. Table S6 provides the sequences for siNAT10. The wild-type and mutated ac4C sites of CXCL5 and DEK were inserted into the pMIR-REPORT vector. You can find the ac4C site sequences in Table S7. In the mutant sequence of CXCL5 and DEK, the C in the ac4C site was substituted with a G. The evaluation of luciferase activity was carried out using the Dual-Luciferase Reporter Assay System (Promega, Wisconsin, USA), adhering to the instructions given by the manufacturer. To achieve the final ratios, the relative luciferase intensity was adjusted based on the Renilla luciferase activity.

### RNA stability analysis

Wild-type and NAT10-knockout A549 cells were placed in 12-well plates and treated with 5 μg mL⁻¹ actinomycin D (Sigma, Missouri, USA) for 0, 3, and 6 h. Afterward, total RNA was collected and examined using qPCR, as previously outlined. The rates of mRNA degradation were determined following the previously documented procedure [47].

### Assessment of cell proliferation

In a 96-well plate, we placed 1 × 10³ cells in each well and grew them for the designated time periods. After treating them with the CCK8 solution (Dojindo, Tokyo, Japan), we measured the absorbance at 450 nm with a microplate reader (Bio-Rad, California, USA) to evaluate how the cells had multiplied.

### Assessment of cell invasion and migration

In the upper chambers of Transwell plates (Corning, New York, USA), 1 × 10^5^ cells were placed, either on Matrigel (BD Biosciences, New Jersey, USA) or on a surface without coating. Following a 48-h incubation period, the cells were treated with paraformaldehyde for fixation and stained using 0.1% crystal violet. A microscope was then used to examine the cells that migrated and invaded, and the cell count was performed in three different fields of view with the help of ImageJ software.

### Assessment of cell adhesion

DMEM, which was treated with various ECM proteins, was incubated at 37 °C for 1 h. The ECM proteins used included collagen I (0.01%, Biosharp, BS929-1g), fibronectin (20 μg/mL, Solarbio, F8180), laminin (0.01%, Solarbio, CLP0114), and poly-(L-lysine) (1 mg/mL, Aladdin, 28211-04-3). After blocking with 0.5% BSA, a total of 2 × 10^4^ cells were introduced into 12-well plates and cultured in DMEM supplemented with 10% FBS for 30 min. Afterwards, the colonies were fixed and stained using 0.1% crystal violet for 10 min, before being imaged and counted for further statistical analysis.

### Dual labeling with FISH and IF staining

Initially, cells were treated with 4% paraformaldehyde for fixation and then subjected to 0.5% Triton X-100 to enhance permeability before being acetylated with triethanolamine for 15 min. Following this, a prehybridization solution was added, and the cells were kept at 65 °C for 1 h. After removing the prehybridization solution, an antisense riboprobe hybridization solution was introduced, and the cells were left to incubate overnight at 65 °C. Next, blocking was performed using 5% BSA, and the cells were kept with anti-Digoxigenin-Fluorescein (1:5000, Roche, 11207741910) in the refrigerator overnight. The cells underwent three washing steps. After being treated with 0.5% Triton X-100 and blocked with 5% BSA, they were incubated overnight at 4 °C with either anti-NAT10 antibody or anti-F-actin antibody (1:50, Abcam, ab205). Following this, secondary antibodies linked to Alexa 488 (1:200, A11034, Thermo Fisher Scientific) or Alexa 568 (1:200, A11036, Thermo Fisher Scientific) were applied and incubated at room temperature for 1 h. Once the cells were washed with PBS, they were mounted with DAPI in the dark. Images were captured using Laser confocal scanning microscopy (Olympus, Japan) for analysis. The primer sequences used for probe synthesis are listed in Table S5. All experimental steps were carried out in a nuclease-free environment.

### Mouse tumor xenograft model

Female nude mice at 4 weeks old were obtained from Charles River and kept in controlled environments free of specific pathogens. We injected 1 × 10⁶ A549 wild-type or NAT10-knockout cells into the tail veins of these nude mice to evaluate their ability to metastasize with no randomization. The weight of the mice was checked every 3 days. After a period of 7 weeks, the mice were euthanized, and their lungs were removed and preserved for immunohistochemistry analysis. We then counted the number of metastatic tumors in the lungs.

### Statistical analysis

The data were reported as mean ± standard deviation. We used GraphPad Prism software to carry out statistical analyses. To compare the differences between groups, both Student’s *T*-test and one-way analysis of variance were applied. A *p* value of less than 0.05 was considered statistically significant in all tests (*p < 0.05; **p < 0.01; ***p < 0.001).

### Permission to publish the figures/graphical abstract in the journal

We have obtained permission to publish the figures/graphical abstract in a journal and ensured it is attributed in the relevant figure legend as “Created in BioRender.com”.

## Supplementary information

supplementary data

Original western blot

aj-checklist

## Data Availability

The raw sequencing data have been deposited in the Gene Expression Omnibus database under the accession number (GSE315123 and GSE315121). All the other data generated in this study are included in the article and the additional files. The datasets generated during and/or analyzed during the current study are available from the corresponding author on reasonable request.
