# Supplementary material for: NAT10-mediated ac4C RNA acetylation stabilizes CXCL5/DEK mRNA to drive proliferation and metastasis in lung adenocarcinoma
Source: Cell Death Dis. 2026 Mar 20;17(1):326. doi: 10.1038/s41419-026-08568-6 (PMC13039259; doi:10.1038/s41419-026-08568-6)
Supplement: Supplementary file 1 — supplementary data [file 41419_2026_8568_MOESM1_ESM.docx]

Figure S1. Using Primer5.0, external primers were designed at both ends of the knockout site to identify the knockout (a) and internal primers were designed at the inner side of the knockout site to identify whether it was a homozygote (b) PCR results showed that the knockout band was 367bp and there was no internal primer to amplify the bands, so we obtained a homozygote clone with NAT10 knockout.


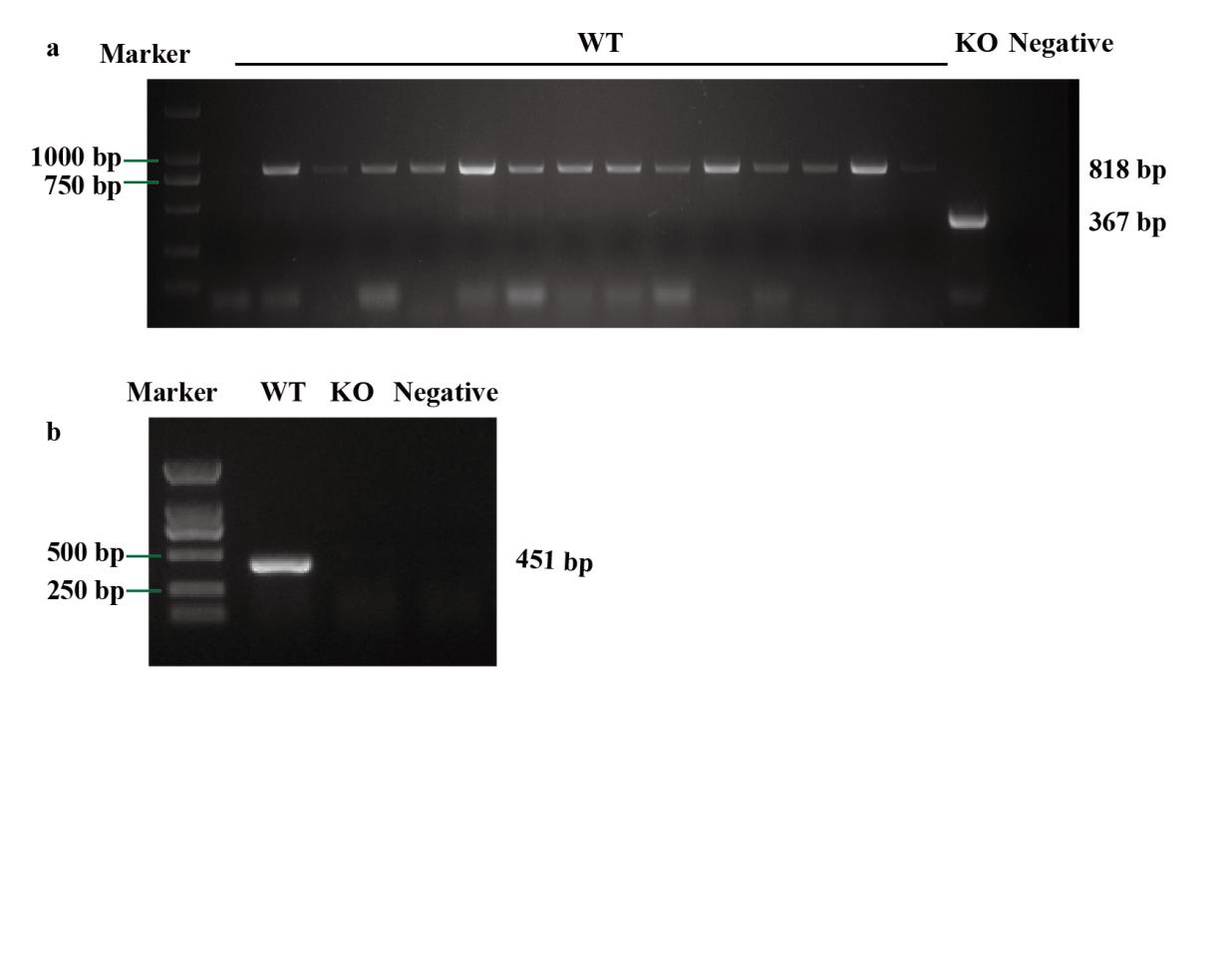


Figure S2. After knocking out NAT10, the enrichment level of ac4C peaks in the coding sequence (CDS) of A549 cells decreased from 39.5% to 22.2% in acRIP-seq data (Figure S2a-b). The most significant downregulation of ac4C modification on chromosome 11 after NAT10 which located on chromosome 11 knockout demonstrated the possibility that NAT10 tends to modify nearby sites. (Figure S2c).


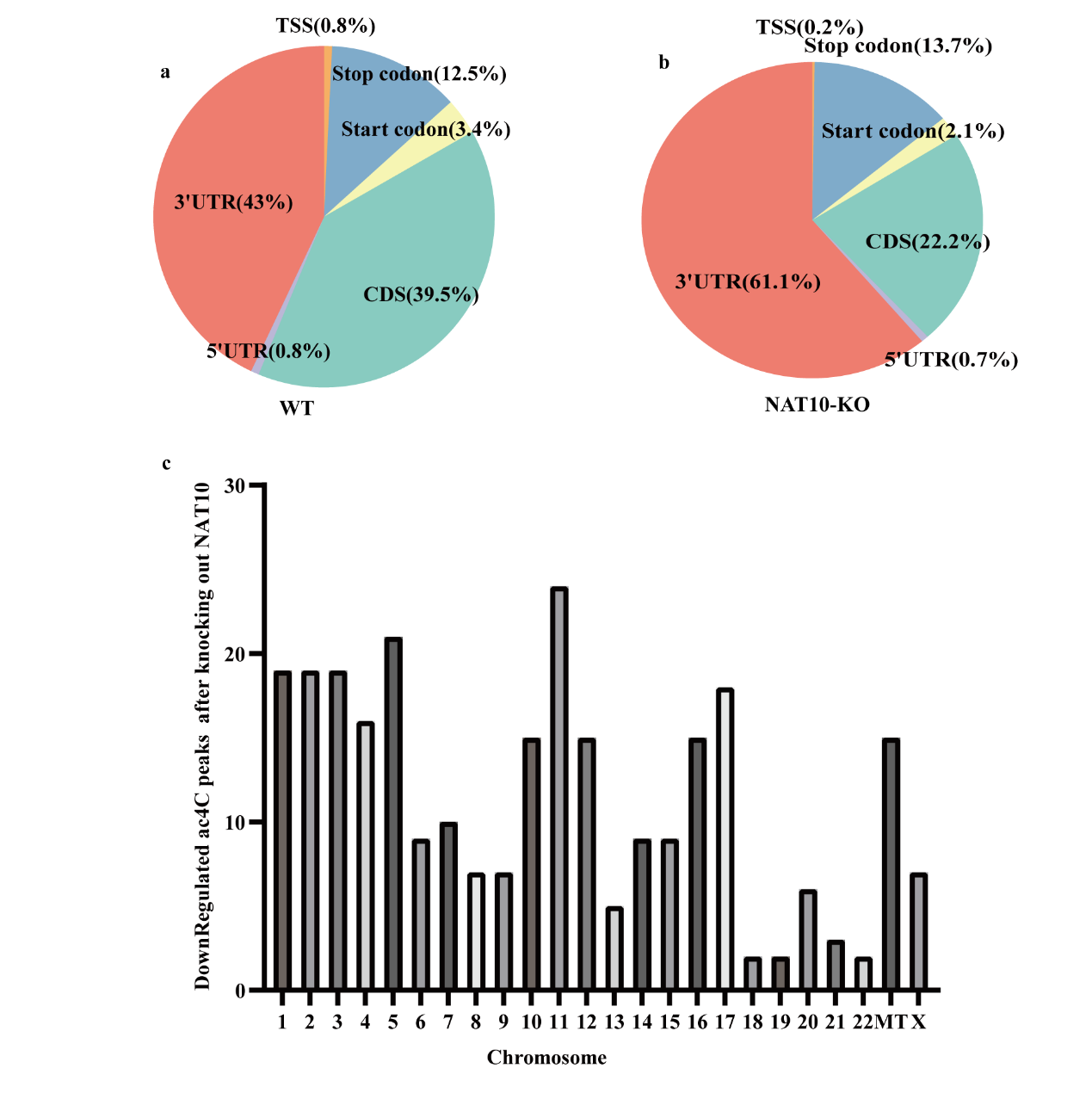


Figure S3. The negative control used a gene without ac4c modification sites, which further proves the reliability of the experiment.

Figure S4. The results indicate that the overexpression of NAT10 can rescue the effects brought about by NAT10 knockout (Figure S4a-c). To further confirm the importance of the acetylation catalytic site of NAT10 in the regulation process of target genes, due to some researches has demonstrated that the G641E site on NAT10 is crucial for catalyzing the acetylation site, we mutated the G641E site on the NAT10 overexpression vector. However, when NAT10-G641E was overexpressed in both wild-type and NAT10 knockout cells, there were no significant changes in the mRNA levels of CXCL5/DEK, which validates the previous researchers' conclusion that NAT10 catalytic activity is crucial for the regulation of target genes (Figure S4d).


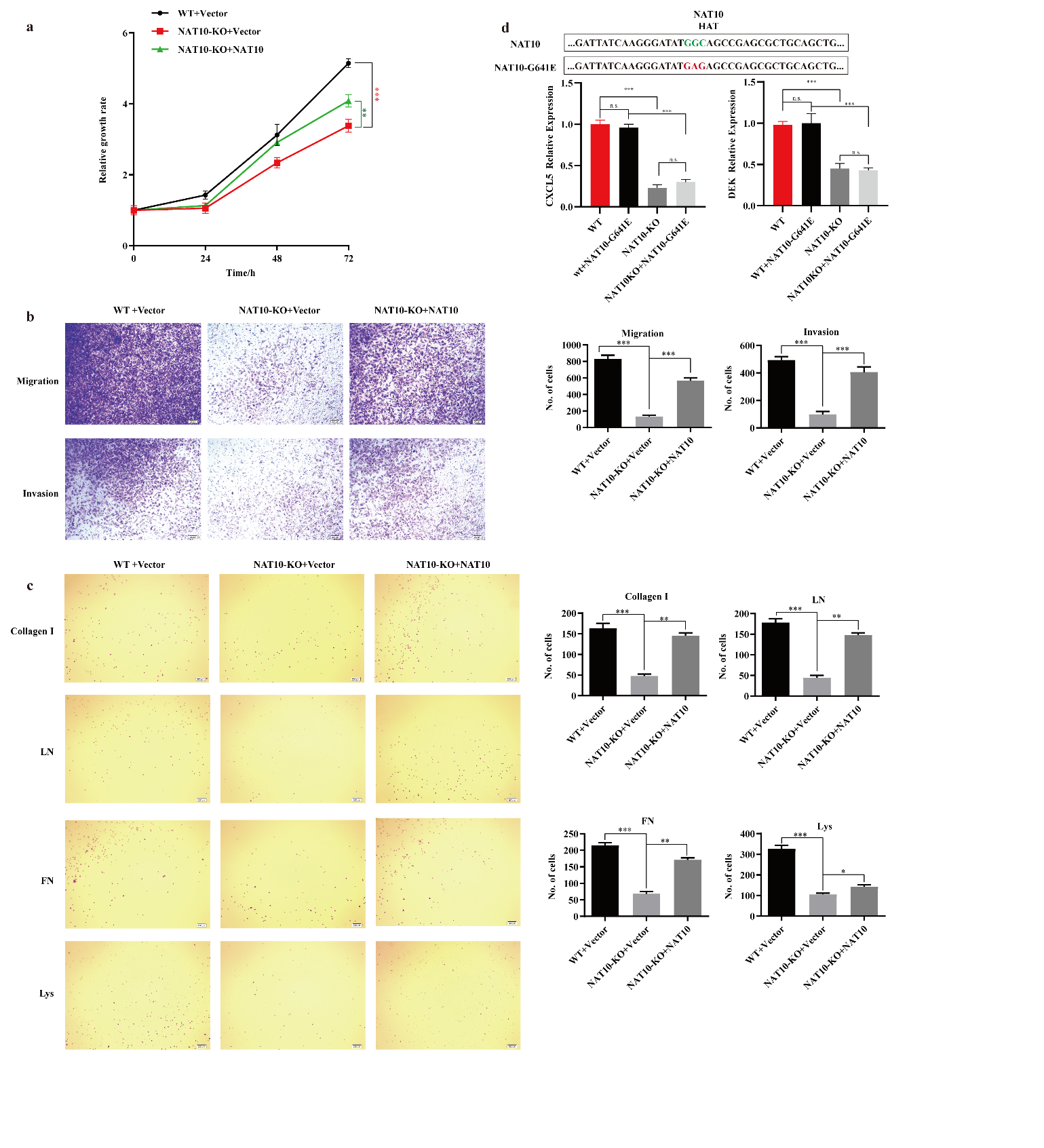


Fig S4. **a** Cell proliferation was measured using the CCK8 assay after NAT10 knockout and NAT10 overexpression. **b** Inhibition of invasion and metastasis of LUAD cells by NAT10 and its rescue effect on NAT10. **c** Inhibition of cell adhesion of LUAD cells by NAT10 and its rescue effect on NAT10**. d** CXCL5 and DEK mRNAs when a catalytic mutant of NAT10 was overexpressed instead. The data in (a) was normalized to WT + Vector levels.

Table S1 qRT-PCR Primer Sequences

| Primer name | Primer sequence |
| --- | --- |
| GAPDH-F | ATGGGGAAGGTGAAGGTCG |
| GAPDH-R | GGGGTCATTGATGGCAACAATA |
| NAT10-F | AGAATGCGACAGCTGCAGAA |
| NAT10-R | TCTACAGTCCTGGCCAGCAA |
| CXCL5-F | AGTGGTAGCCTCCCTGAAGAAC |
| CXCL5-R | CTTCTCTGCTGAAGACTGGGAA |
| DEK-F | GTCCGAGAAAGAACCCGAA |
| DEK-R | TTTCCCTCTTGCCTTCCAC |

Table S2 CRISPR-Cas9 relative Primer Sequences

| Primer name | Primer sequence |
| --- | --- |
| hNAT10-KO-818 bp-F | GCCTGAATAATGACTCCTGTGA |
| hNAT10-KO-818 bp-R | CAGAGGCTGTGCTGACTACT |
| hNAT10-KO-451 bp-F | GCCTGAATAATGACTCCTGTGA |
| hNAT10-KO-451 bp-R | CAGAGGCTGTGCTGACTACT |
| BbsI-Off-Target-1 F | CCGTTGGTAGACCTATCATTCCA |
| BbsI-Off-Target-1 R | AACCCGTATTCTGCTCCATCT |
| BbsI-Off-Target-2 F | CAACCTCCAGGTGAGGATCA |
| BbsI-Off-Target-2 R | CGGAGAAGATTCGACCTCCC |
| BsaI-Off-Target-1 F | AATGGGGCGCATCTCTCATT |
| BsaI-Off-Target-1 R | TCCCCTTTTCTTCTGGGCAC |
| BsaI-Off-Target-2 F | GTGGCTCTCAACTTCGGCT |
| BsaI-Off-Target-2 R2 | GAATGCCACGCTTGATCCTT |

Table S3 107 Genes whose expression decreased significantly after NAT10 knocking out (log₂Fold Change of under -1 and a *p*-value lower than 0.05)

| Ensembl Gene ID | | | |
| --- | --- | --- | --- |
| ENSG00000133392 | ENSG00000263065 | ENSG00000175084 | ENSG00000072864 |
| ENSG00000149591 | ENSG00000110195 | ENSG00000083168 | ENSG00000249915 |
| ENSG00000182858 | ENSG00000070018 | ENSG00000099797 | ENSG00000263335 |
| ENSG00000230551 | ENSG00000141424 | ENSG00000214021 | ENSG00000101901 |
| ENSG00000112983 | ENSG00000138778 | ENSG00000125107 | ENSG00000162896 |
| ENSG00000138756 | ENSG00000029725 | ENSG00000203950 | ENSG00000067334 |
| ENSG00000138802 | ENSG00000120875 | ENSG00000105939 | ENSG00000249669 |
| ENSG00000100664 | ENSG00000113712 | ENSG00000122779 | ENSG00000175582 |
| ENSG00000179222 | ENSG00000117983 | ENSG00000069275 | ENSG00000123684 |
| ENSG00000157540 | ENSG00000115464 | ENSG00000250031 | ENSG00000283010 |
| ENSG00000101335 | ENSG00000095637 | ENSG00000152484 | ENSG00000140395 |
| ENSG00000165650 | ENSG00000205765 | ENSG00000039523 | ENSG00000060138 |
| ENSG00000167637 | ENSG00000163735 | ENSG00000117906 | ENSG00000074527 |
| ENSG00000125977 | ENSG00000151176 | ENSG00000133816 | ENSG00000167522 |
| ENSG00000168631 | ENSG00000178429 | ENSG00000106400 | ENSG00000038219 |
| ENSG00000162512 | ENSG00000172403 | ENSG00000138069 | ENSG00000099194 |
| ENSG00000106993 | ENSG00000006453 | ENSG00000111229 | ENSG00000133112 |
| ENSG00000259201 | ENSG00000011426 | ENSG00000102543 | ENSG00000119125 |
| ENSG00000148344 | ENSG00000129226 | ENSG00000127947 | ENSG00000131462 |
| ENSG00000116750 | ENSG00000113558 | ENSG00000104067 | ENSG00000180817 |
| ENSG00000173812 | ENSG00000164244 | ENSG00000137831 | ENSG00000162298 |
| ENSG00000177707 | ENSG00000100504 | ENSG00000163017 | ENSG00000163132 |
| ENSG00000147853 | ENSG00000144674 | ENSG00000251705 | ENSG00000089775 |
| ENSG00000118363 | ENSG00000124795 | ENSG00000115756 | ENSG00000023171 |
| ENSG00000273192 | ENSG00000071537 | ENSG00000047410 | ENSG00000005339 |
| ENSG00000141956 | ENSG00000283782 | ENSG00000284681 | ENSG00000142920 |
| ENSG00000264772 | ENSG00000135801 | ENSG00000049618 |  |

Table S4 199 Genes whose expression decreased significantly after NAT10 knocking out (log₂Fold Change of under 0 and a *p*-value lower than 0.05)

| Ensembl Gene ID | | | |
| --- | --- | --- | --- |
| ENSG00000133392 | ENSG00000175084 | ENSG00000072864 | ENSG00000163017 |
| ENSG00000149591 | ENSG00000110195 | ENSG00000083168 | ENSG00000249915 |
| ENSG00000147853 | ENSG00000182858 | ENSG00000070018 | ENSG00000099797 |
| ENSG00000118363 | ENSG00000141424 | ENSG00000214021 | ENSG00000101901 |
| ENSG00000112983 | ENSG00000138778 | ENSG00000125107 | ENSG00000162896 |
| ENSG00000141956 | ENSG00000138756 | ENSG00000029725 | ENSG00000203950 |
| ENSG00000067334 | ENSG00000138802 | ENSG00000120875 | ENSG00000105939 |
| ENSG00000115756 | ENSG00000100664 | ENSG00000113712 | ENSG00000122779 |
| ENSG00000175582 | ENSG00000047410 | ENSG00000179222 | ENSG00000117983 |
| ENSG00000069275 | ENSG00000123684 | ENSG00000284681 | ENSG00000157540 |
| ENSG00000115464 | ENSG00000163132 | ENSG00000101335 | ENSG00000095637 |
| ENSG00000152484 | ENSG00000140395 | ENSG00000089775 | ENSG00000165650 |
| ENSG00000205765 | ENSG00000039523 | ENSG00000060138 | ENSG00000023171 |
| ENSG00000167637 | ENSG00000163735 | ENSG00000117906 | ENSG00000074527 |
| ENSG00000005339 | ENSG00000125977 | ENSG00000151176 | ENSG00000133816 |
| ENSG00000167522 | ENSG00000142920 | ENSG00000168631 | ENSG00000106400 |
| ENSG00000038219 | ENSG00000283782 | ENSG00000162512 | ENSG00000172403 |
| ENSG00000138069 | ENSG00000099194 | ENSG00000135801 | ENSG00000106993 |
| ENSG00000006453 | ENSG00000111229 | ENSG00000133112 | ENSG00000011426 |
| ENSG00000102543 | ENSG00000119125 | ENSG00000049618 | ENSG00000148344 |
| ENSG00000129226 | ENSG00000127947 | ENSG00000131462 | ENSG00000144674 |
| ENSG00000116750 | ENSG00000113558 | ENSG00000104067 | ENSG00000180817 |
| ENSG00000124795 | ENSG00000173812 | ENSG00000164244 | ENSG00000137831 |
| ENSG00000162298 | ENSG00000071537 | ENSG00000177707 | ENSG00000100504 |
| ENSG00000111371 | ENSG00000196865 | ENSG00000148773 | ENSG00000198646 |
| ENSG00000108654 | ENSG00000108256 | ENSG00000173402 | ENSG00000112378 |
| ENSG00000117523 | ENSG00000107863 | ENSG00000167193 | ENSG00000108055 |
| ENSG00000108848 | ENSG00000163682 | ENSG00000060339 | ENSG00000138182 |
| ENSG00000180530 | ENSG00000167088 | ENSG00000171345 | ENSG00000280987 |
| ENSG00000124486 | ENSG00000184258 | ENSG00000056097 | ENSG00000153113 |
| ENSG00000198695 | ENSG00000198393 | ENSG00000026508 | ENSG00000112851 |
| ENSG00000070961 | ENSG00000100644 | ENSG00000147274 | ENSG00000105879 |
| ENSG00000109971 | ENSG00000198763 | ENSG00000185621 | ENSG00000135486 |
| ENSG00000228253 | ENSG00000196504 | ENSG00000198900 | ENSG00000169908 |
| ENSG00000168439 | ENSG00000117724 | ENSG00000180917 | ENSG00000198888 |
| ENSG00000198899 | ENSG00000198786 | ENSG00000172239 | ENSG00000213186 |
| ENSG00000120837 | ENSG00000131711 | ENSG00000137819 | ENSG00000182253 |
| ENSG00000172115 | ENSG00000105974 | ENSG00000136450 | ENSG00000167978 |
| ENSG00000137845 | ENSG00000198727 | ENSG00000127022 | ENSG00000114346 |
| ENSG00000132763 | ENSG00000184743 | ENSG00000198938 | ENSG00000270757 |
| ENSG00000198242 | ENSG00000152795 | ENSG00000143621 | ENSG00000212907 |
| ENSG00000144713 | ENSG00000043093 | ENSG00000138398 | ENSG00000116489 |
| ENSG00000123200 | ENSG00000257529 | ENSG00000198886 | ENSG00000153147 |
| ENSG00000067167 | ENSG00000171067 | ENSG00000116560 | ENSG00000067064 |
| ENSG00000181163 | ENSG00000141232 | ENSG00000100941 | ENSG00000198804 |
| ENSG00000122406 | ENSG00000163806 | ENSG00000108953 | ENSG00000113013 |
| ENSG00000185787 | ENSG00000115053 | ENSG00000129116 | ENSG00000125378 |
| ENSG00000096384 | ENSG00000163931 | ENSG00000137309 | ENSG00000255508 |
| ENSG00000178988 | ENSG00000143624 | ENSG00000170385 | ENSG00000204628 |
| ENSG00000008988 | ENSG00000134294 | ENSG00000140374 |  |

Table S5 ac4C relative Sequences

| Primer name | Primer sequence |
| --- | --- |
| CXCL5-site1-3-F | GCTGCTGCTGCTGCTGAC |
| CXCL5-site1-3-R | GCGTGGTCTGTAAACAAACG |
| CXCL5-site4-F | GCCGCTTAAGCTTTCAGCTC |
| CXCL5-site4-R | TGCCAAAACTTCAATAGCATAGCA |
| DEK-ac4C site-F | CCACCAAAAAAGACAGCCAAAAGAG |
| DEK-ac4C site-R | CTTTTTTGGAACTGTTTTGATTCTTCTTGGT |
| DEK-non ac4C site-F | CCATTGCCGAAATCTAAAAAAACTTGTAG |
| DEK-non ac4C site-R | GACTCTTCCTTGTTTTTCTTTTCATCTTCA |
| CXCL5-FISH-F | CCCAAGCTTCTCTCTTGACCACTATGAG |
| CXCL5-FISH-R | CCCAAGCTTTAAAATAACAGCAAATAGC |
| DEK-FISH-F | CCCAAGCTTAAAAGAGAAAAACCTAAAC |
| DEK-FISH-R | CCCAAGCTTTTTTACAGTTGTTTTTATG |

Table S6 SiRNA-Sequences

| Si-Name | Si- Sequence |
| --- | --- |
| si-NC-sense | UUCUCCGAACGUGUCACGUTT |
| si-NC-antisense | ACGUGACACGUUCGGAGAATT |
| si-NAT10-sense | AGUGGAAGGUGGUGGGCUAUU |
| si-NAT10-antisense | UAGCCCACCACCUUCCACUUU |

Table S7 Sequences

| Name | Sequence |
| --- | --- |
| CXCL5-site1-3-WT | GCTGCTGCTGCTGCTGACGCAGCCAGGGCCCATCGCCAGCGCTGGTCCTGCCGCTGCTGTGTTGAGAGAGCTGCGTTGCGTTTGTTTACAGACCACGC |
| CXCL5-site1-3-MUT | GGTGGTGGTGGTGGTGAGGGAGGGAGGGGGGATGGGGAGGGGTGGTGGTGGGGGTGGTGTGTTGAGAGAGGTGGGTTGGGTTTGTTTAGAGAGGAGGG |
| CXCL5-site4-WT | GCCGCTTAAGCTTTCAGCTCAGCTAATGAAGTGTTTAGCATAGTACCTCTGCTATTTGCTGTTATTTTATCTGCTATGCTATTGAAGTTTTGGCA |
| CXCL5-site4-MUT | GGGGGTTAAGGTTTGAGGTGAGGTAATGAAGTGTTTAGGATAGTAGGTGTGGTATTTGGTGTTATTTTATGTGGTATGGTATTGAAGTTTTGGGA |
| DEK-ac4C site-WT | CCACCAAAAAAGACAGCCAAAAGAGAAAAACCTAAACAGAAAGCTACTTCTAAAAGTAAAAAATCTGTGAAAAGTGCCAATGTTAAGAAAGCAGATAGCAGCACCACCAAGAAGAATCAAAACAGTTCCAAAAAAG |
| DEK-ac4C site-MUT | GGAGGAAAAAAGAGAGGGAAAAGAGAAAAAGGTAAAGAGAAAGGTAGTTGTAAAAGTAAAAAATGTGTGAAAAGTGGGAATGTTAAGAAAGGAGATAGGAGGAGGAGGAAGAAGAATGAAAAGAGTTGGAAAAAAG |
| DEK-non ac4C site-WT | CCATTGCCGAAATCTAAAAAAACTTGTAGCAAAGGCAGTAAAAAGGAACGGAACAGTTCTGGAATGGCAAGGAAGGCTAAGCGAACCAAATGTCCTGAAATTCTGTCAGATGAATCTAGTAGTGATGAAGATGAAAAGAAAAACAAGGAAGAGTC |
| DEK-non ac4C site-MUT | GGATTGGGGAAATGTAAAAAAAGTTGTAGGAAAGGGAGTAAAAAGGAAGGGAAGAGTTGTGGAATGGGAAGGAAGGGTAAGGGAAGGAAATGTGGTGAAATTGTGTGAGATGAATGTAGTAGTGATGAAGATGAAAAGAAAAAGAAGGAAGAGTG |
