# Supplementary figures and images for: NAT10-mediated ac4C RNA acetylation stabilizes CXCL5/DEK mRNA to drive proliferation and metastasis in lung adenocarcinoma
Source: Cell Death Dis. 2026 Mar 20;17(1):326. doi: 10.1038/s41419-026-08568-6 (PMC13039259; doi:10.1038/s41419-026-08568-6)

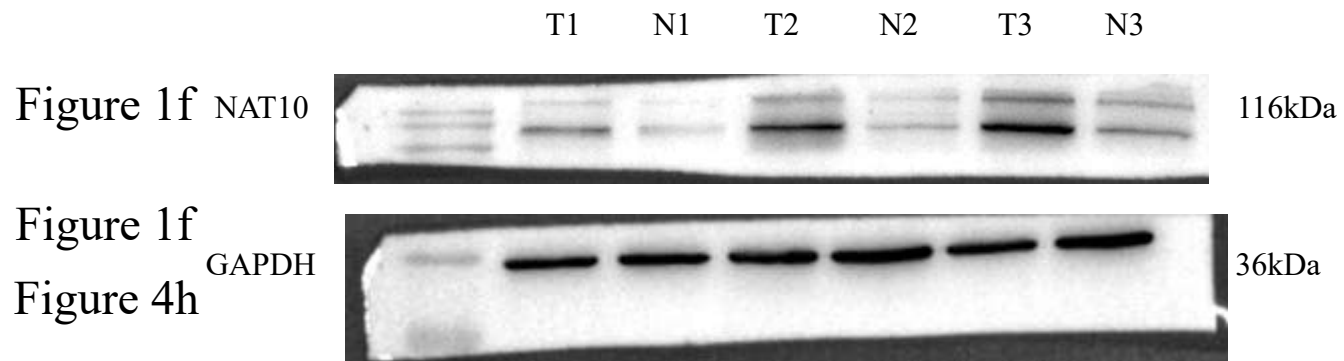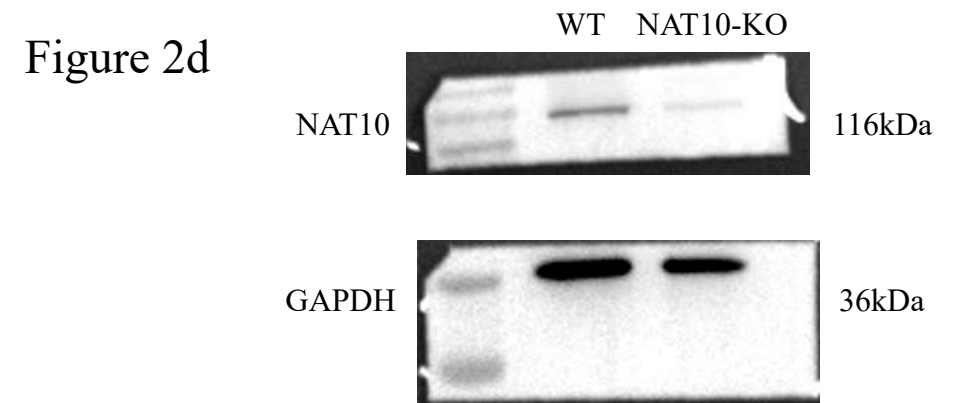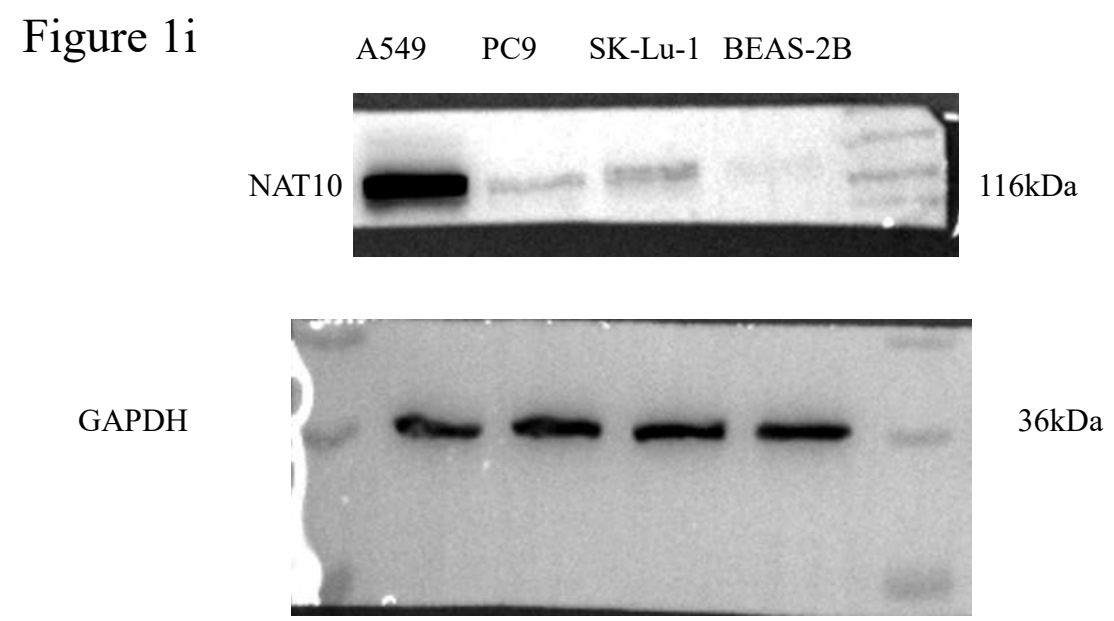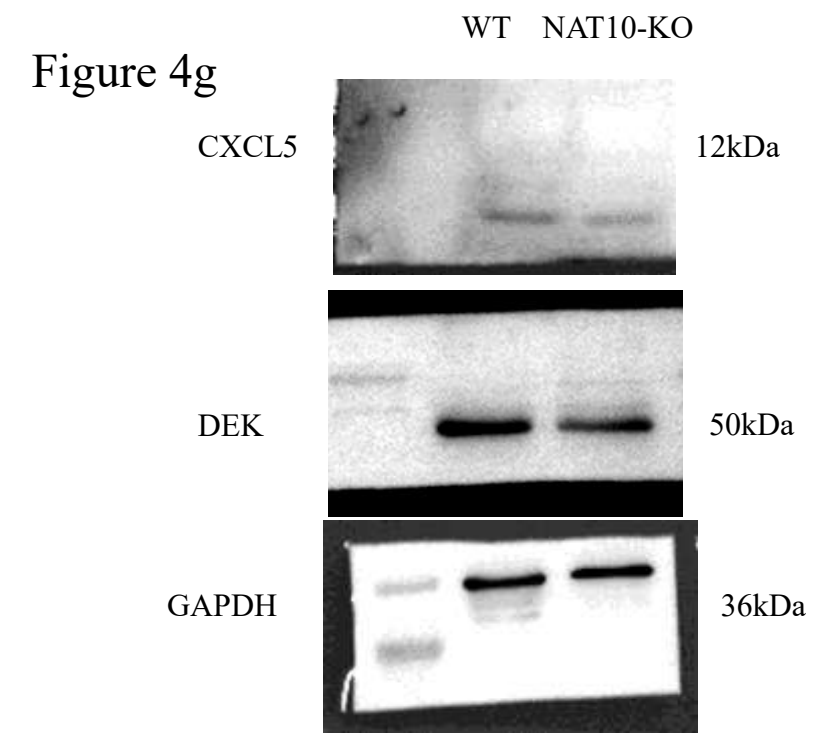

Figure 4h

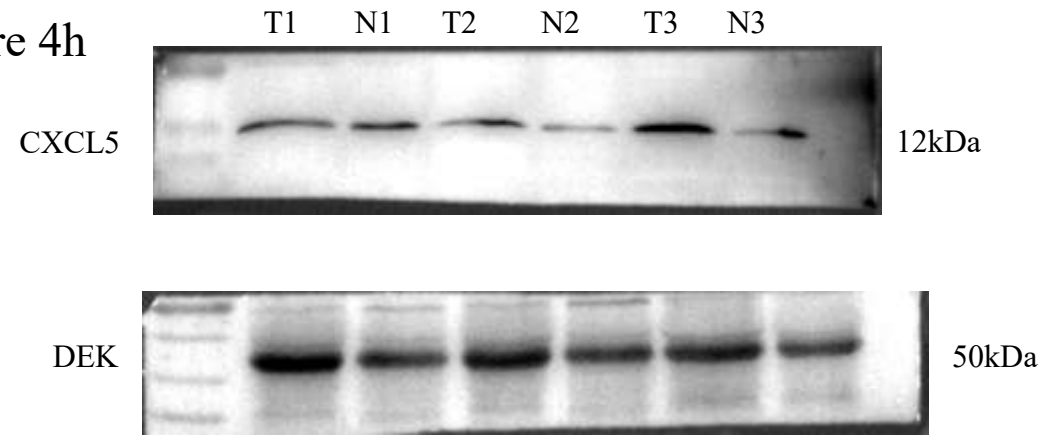

Figure 5c

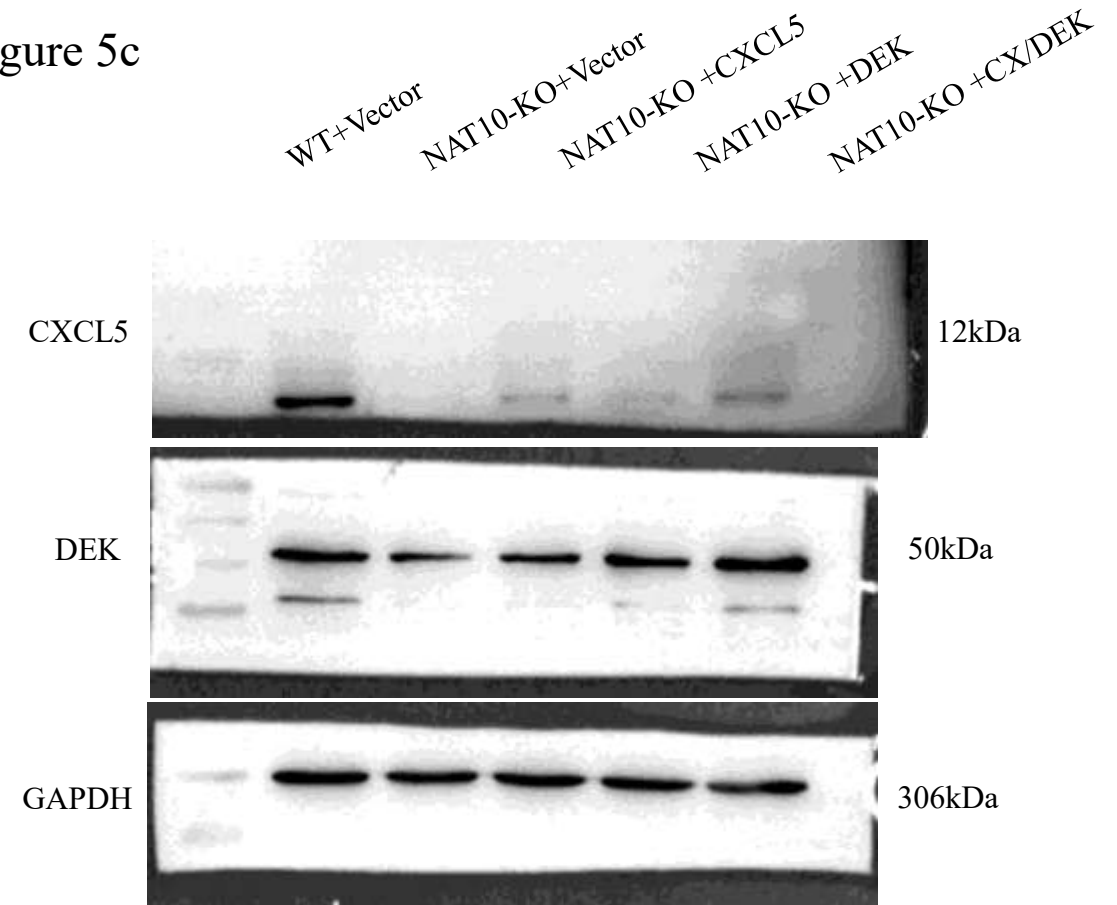

Supplement: Supplementary file 2 — Original western blot [file 41419_2026_8568_MOESM2_ESM.pdf]
